# Statistical Analysis for Competing Risks' Model with Two Dependent Failure Modes from Marshall–Olkin Bivariate Gompertz Distribution

**DOI:** 10.1155/2022/3988225

**Published:** 2022-05-28

**Authors:** Min Wu, Fode Zhang, Yimin Shi, Yan Wang

**Affiliations:** ^1^School of Economics and Management, Shanghai Maritime University, Shanghai 201306, China; ^2^Center of Statistical Research, School of Statistics, Southwestern University of Finance and Economics, Chengdu 611130, Sichuan, China; ^3^School of Mathematics and Statistics, Northwestern Polytechnical University, Xi'an 710072, Shaanxi, China; ^4^School of Science, Xi'an Polytechnic University, Xi'an 710072, Shaanxi, China

## Abstract

The bivariate or multivariate distribution can be used to account for the dependence structure between different failure modes. This paper considers two dependent competing failure modes from Gompertz distribution, and the dependence structure of these two failure modes is handled by the Marshall–Olkin bivariate distribution. We obtain the maximum likelihood estimates (MLEs) based on classical likelihood theory and the associated bootstrap confidence intervals (CIs). The posterior density function based on the conjugate prior and noninformative (Jeffreys and Reference) priors are studied; we obtain the Bayesian estimates in explicit forms and construct the associated highest posterior density (HPD) CIs. The performance of the proposed methods is assessed by numerical illustration.

## 1. Introduction

It is extremely common that the failure of a product or a system contains several competing failure modes in reliability engineering; any failure mode will lead to the failure result. Competing risks' data contain the failure time and the corresponding failure mode, which can be modeled by the competing risks' model and has been commonly performed in many research fields, such as engineering and medical statistics. Previous studies have mostly assumed the competing failure modes to be independent; Wang et al. [1], Ren and Gui [2], and Qin and Gui [3] focused on the independent competing risks' model under progressively hybrid censoring from Weibull and Burr-XII distributions. Objective Bayesian analysis for the competing risks' model with Wiener degradation phenomena and catastrophic failures was studied by Guan et al. [4]. In practice, the independency relationship between different failure modes is a very special case; a more common situation is dependency. That is, the failure mechanisms are interactive and interdependent; the occurrence of one failure mode will affect the occurrence of other failure modes. For example, a ship fixed carbon dioxide fire extinguishing system can fail due to pressure gauge, distribution value, cylinder group, and so on; these failure modes are dependent because they all are related to the storage environment. Therefore, it is more reasonable to assume dependency among different competing failure modes. The competing risks' model considers the product or system with multiple dependent competing failure modes, any one of which will cause the occurrence of failure. The dependent competing risks' model has been extensively studied. Zhang et al. [5] and Zhang et al. [6] studied the dependent competing risks' model under accelerated life testing (ALT) by copula function to measure the dependence between different competing failure modes; the results indicate the copula construction method has good accuracy and universality. Wang and Yan [7] and Wu et al. [8] also studied this model under ALT and progressively hybrid-censoring scheme using Clayton copula and Gumbel copula, respectively. For other related works, see the works of Lo and Wike [9] and Fang et al. [10].

In addition to using copula function to handle the relationship between different competing failure modes, the bivariate or multivariate distribution also can be used to account for the correlation between different failure modes. The Marshall–Olkin distribution [11], which has many good properties, is the best-known bivariate distribution and has been discussed extensively; it has a parameter to describe the dependence structure. Li et al. [12], Kundu and Gupta [13], and Bai et al. [14] provided reviews on Marshall–Olkin–Weibull distribution; Kundu and Gupta [13] obtained the explicit forms of the unknown parameters when the shape parameter is known; when the shape parameter is unknown, they used the importance sampling to compute the Bayesian estimates of the unknown parameters. Bai et al. [14] discussed the statistical analysis for the accelerated dependent competing risks' model under Type-II hybrid censoring schemes. Guan et al. [15] studied objective Bayesian analysis for the Marshall–Olkin exponential distribution based on reference priors; they also found that some of the reference priors are also matching priors and the posterior distributions based on these priors are proper.

The Gompertz distribution is a widely used growth model which has been studied extensively; Ismail [16] studied the Bayesian analysis of Gompertz distribution parameters and acceleration factor in the case of partially accelerated life testing under Type-I censoring scheme. Ghitany et al. [17] considered a progressively censored sample from Gompertz distribution; they discussed the existence and uniqueness of the MLEs of the unknown parameters. The Gompertz distribution plays an important role in fitting clinical trials' data in medical science and can be used to the theory of extreme-order statistics. In this paper, we will study the dependent competing risks' model from the Marshall–Olkin bivariate Gompertz (MOGP) distribution, which is a bivariate distribution with Gompertz marginal distributions. We focus our attention on the statistical analysis of the model parameters, including classical likelihood inference, Bayesian analysis, and objective Bayesian analysis. Because the Bayesian analysis based on conjugate prior is sensitive to the hyperparameters, inappropriate choice of it will cause bad priors. Based on this reason, we propose the objective Bayesian analysis based on noninformative priors for comparison. The objective Bayesian inference has been studied by Guan et al. [14], Bernardo [18], and Berger and Bernardo [19] based on Reference and Jeffreys priors.

In the rest of this paper, we will present the model description and some properties.  Section 3 presents the MLEs and associated bootstrap CIs. In Section 4, Bayesian estimates and associated HPD CIs based on conjugate prior, Jeffreys prior [20], and reference priors [18] are obtained, and these priors lead to proper posteriors which are proved.  Section 5 presents some results obtained from simulation study and illustrative analysis.  Section 6 gives some final concluding remarks.

## 2. Model Description

Suppose that *f*(*t*; *λ*, *θ*) is a Gompertz distribution; the density function and reliability function of it are

(1) $$\begin{array}{c} f\left(t;\lambda ,\theta \right)=\theta e^{\left(\lambda t-\theta \right.\left.\left(e^{\lambda t}-1\right)/\lambda \right)},\;\lambda ,\;\theta >0,\;t>0,\\ S\left(t;\lambda ,\theta \right)=e^{\left(-\theta \right.\left.\left(e^{\lambda t}-1\right)/\lambda \right)},\;\lambda ,\;\theta >0,\;t>0, \end{array}$$

where *λ* is shape parameter and *θ* is scale parameter.

Suppose *U*_0_,  *U*_1_,  and  *U*_2_ are three independent Gompertz variables with different scale parameters, that is, *U*_0_ ~ *GP*(*λ*, *θ*_0_), *U*_1_ ~ *GP*(*λ*, *θ*_1_), and *U*_2_ ~ *GP*(*λ*, *θ*_2_). Let *T*_1_=min(*U*_0_, *U*_1_) and *T*_2_=min(*U*_0_, *U*_2_); we obtain *T*_1_ ~ *GP*(*λ*, *θ*_0_+*θ*_1_) and *T*_2_ ~ *GP*(*λ*, *θ*_0_+*θ*_2_). Then, the pair of variables (*T*_1_, *T*_2_) follows the MOGP distribution denoted by (*T*_1_, *T*_2_) ~ MOGP(*λ*, *θ*_0_, *θ*_1_, *θ*_2_). When *θ*_0_=0, the two variables *T*_1_ and *T*_2_ are independent and *T*_1_ and *T*_2_ will be dependent when *θ*_0_ > 0; hence, *θ*_0_ can be regarded as a correlation coefficient between *T*_1_ and *T*_2_.

The joint PDF of (*T*_1_,  *T*_2_) can be written as

(2) $$\begin{array}{c} f_{T_{1},\;T_{2}}\left(t_{1},t_{2};\lambda ,\theta_{0},\theta_{1},\theta_{2}\right)=\left\{\begin{array}{cc} f\left(t_{1};\lambda ,\theta_{0}+\theta_{1}\right)f\left(t_{2};\lambda ,\theta_{2}\right) & t_{1}>t_{2}\\ f\left(t_{1};\lambda ,\theta_{1}\right)f\left(t_{2};\lambda ,\theta_{0}+\theta_{2}\right) & t_{1}<t_{2}.\\ \left(\frac{\theta_{0}}{\left(\theta_{0}+\theta_{1}+\theta_{2}\right)}\right)f\left(t;\lambda ,\theta_{0}+\theta_{1}+\theta_{2}\right) & t_{1}=t_{2}=t \end{array}\right.. \end{array}$$

**Figure 1:**
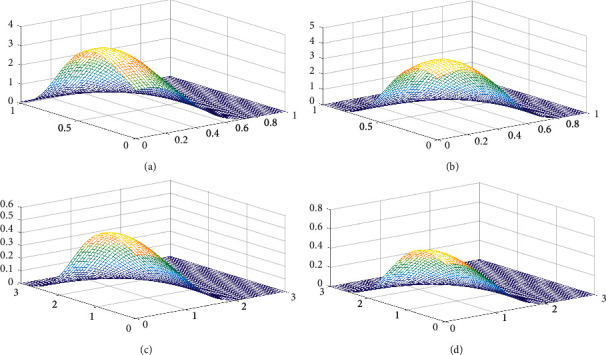
Surface plot of *f*_*T*_1_, *T*_2__(*t*_1_, *t*_2_; *λ*, *θ*_0_, *θ*_1_, *θ*_2_) with different values of *λ*,  *θ*_0_,  *θ*_1_,  *θ*_2_. (a) (*λ*,  *θ*_0_,  *θ*_1_,  *θ*_2_)=(3,  0.5,  2,  1). (b) (*λ*,  *θ*_0_,  *θ*_1_,  *θ*_2_)=(3,  1.5,  0.5,  2). (c) (*λ*,  *θ*_0_,  *θ*_1_,  *θ*_2_)=(1,  0.5,  0.5,  0.5). (d) (*λ*,  *θ*_0_,  *θ*_1_,  *θ*_2_)=(1,  0.2,  0.8,  0.6).

The surface plots of *f*_*T*_1_, *T*_2__(*t*_1_, *t*_2_; *λ*, *θ*_0_, *θ*_1_, *θ*_2_) are presented in Figure 1. From Figure 1, we can see that *f*_*T*_1_, *T*_2__(*t*_1_, *t*_2_; *λ*, *θ*_0_, *θ*_1_, *θ*_2_) is a unimodal function.

Put *n* identical products into test, and each product has two dependent failure modes with lifetimes *T*_1_,  *T*_2_, (*T*_1_, *T*_2_) ~ MOGP(*λ*, *θ*_0_, *θ*_1_, *θ*_2_). Then, the system lifetime is *X*=min(*T*_1_, *T*_2_) ~ MOGP(*λ*, *θ*_0_+*θ*_1_+*θ*_2_). Let *δ*_0*l*_=*I*(*T*_1*l*_=*T*_2*l*_), *δ*_1*l*_=*I*(*T*_1*l*_ < *T*_2*l*_), and *δ*_2*l*_=*I*(*T*_1*l*_ > *T*_2*l*_), for *l*=1, ⋯, *n*, where *I*(·) is an indicator function. Then, we can compute *n*_0_=∑_*l*_*δ*_0*l*_, *n*_1_=∑_*l*_*δ*_1*l*_,  *n*_2_=∑_*l*_*δ*_2*l*_, and *n*=*n*_0_+*n*_1_+*n*_2_.

**Theorem 1 .**

For *l*=1, ⋯, *n*, *δ*_0*l*_=*I*(*T*_1*l*_=*T*_2*l*_), *δ*_1*l*_=*I*(*T*_1*l*_ < *T*_2*l*_), and *δ*_2*l*_=*I*(*T*_1*l*_ > *T*_2*l*_), We have

(3) $$\begin{array}{c} \left(\delta_{\text{0}l},\delta_{1l},\delta_{2l}\right)∼\text{Multinomial}\left(1;\frac{\theta_{0}}{\theta_{0}+\theta_{1}+\theta_{2}},\frac{\theta_{1}}{\theta_{0}+\theta_{1}+\theta_{2}},\frac{\theta_{2}}{\theta_{0}+\theta_{1}+\theta_{2}}\right),l=1,\cdots ,n. \end{array}$$

**Proof**

For *l*=1, ⋯, *n*, we have *δ*_0*l*_+*δ*_1*l*_+*δ*_2*l*_=1,

(4) $$\begin{array}{c} P\left(T_{1}<T_{2}\right)=\int_{0}^{\infty }\int_{0}^{t_{2}}f\left(t_{1};\lambda ,\theta_{1}\right)f\left(t_{2};\lambda ,\theta_{0}+\theta_{2}\right){\text{dt}}_{1}{\text{dt}}_{2}=\frac{\theta_{1}}{\theta_{0}+\theta_{1}+\theta_{2}},\\ P\left(T_{1}>T_{2}\right)=\int_{0}^{\infty }\int_{0}^{t_{1}}f\left(t_{1};\lambda ,\theta_{0}+\theta_{1}\right)f\left(t_{2};\lambda ,\theta_{2}\right){\text{dt}}_{2}{\text{dt}}_{1}=\frac{\theta_{2}}{\theta_{0}+\theta_{1}+\theta_{2}},\\ P\left(T_{1}=T_{2}\right)=1-\frac{\theta_{1}}{\theta_{0}+\theta_{1}+\theta_{2}}-\frac{\theta_{2}}{\theta_{0}+\theta_{1}+\theta_{2}}=\frac{\theta_{0}}{\theta_{0}+\theta_{1}+\theta_{2}}. \end{array}$$

Therefore, (*δ*_0*l*_, *δ*_1*l*_, *δ*_2*l*_) ~ Multinomial(1; *θ*_0_/(*θ*_0_+*θ*_1_+*θ*_2_), *θ*_1_/(*θ*_0_+*θ*_1_+*θ*_2_), *θ*_2_/(*θ*_0_+*θ*_1_+*θ*_2_)).

The likelihood function is

(5) $$\begin{array}{c} L\left(\lambda ,\theta_{0},\theta_{1},\theta_{2}\right)=\underset{l}{\prod }{\left[f_{T_{1},\;T_{2}}\left(x_{l},x_{l};\lambda ,\theta_{0},\theta_{1},\theta_{2}\right)\right]}^{\delta_{0l}}{\left[-\frac{\partial S_{T_{1},\;T_{2}}\left(t_{1},t_{2};\lambda ,\theta_{0},\theta_{1},\theta_{2}\right)}{\partial t_{1}}|{}_{\left(x_{l},\;x_{l}\right)}\right]}^{\delta_{1l}},\\ \times {\left[-{\frac{\partial S_{T_{1},\;T_{2}}\left(t_{1},t_{2};\lambda ,\theta_{0},\theta_{1},\theta_{2}\right)}{\partial t_{2}}}_{|\left(x_{l},\;x_{l}\right)}\right]}^{\delta_{2l}}, \end{array}$$

where

(6) $$\begin{array}{c} f_{T_{1},\;T_{2}}\left(x_{l},x_{l};\lambda ,\theta_{0},\theta_{1},\theta_{2}\right)=\frac{\theta_{0}}{\theta_{0}+\theta_{1}+\theta_{2}}f\left(t;\lambda ,\theta_{0}+\theta_{1}+\theta_{2}\right),\\ =\theta_{0}\exp\left\{\lambda x_{l}-\frac{\theta_{0}+\theta_{1}+\theta_{2}}{\lambda }\left(e^{\lambda x_{l}}-1\right)\right\},\\ -\frac{\partial S_{T_{1},\;T_{2}}\left(t_{1},t_{2};\lambda ,\theta_{0},\theta_{1},\theta_{2}\right)}{\partial t_{1}}|{}_{\left(x_{l},\;x_{l}\right)},\\ =\theta_{1}\exp\left\{\lambda x_{l}-\frac{\theta_{0}+\theta_{1}+\theta_{2}}{\lambda }\left(e^{\lambda x_{l}}-1\right)\right\},\\ -\frac{\partial S_{T_{1},T_{2}}\left(t_{1},t_{2};\lambda ,\theta_{0},\theta_{1},\theta_{2}\right)}{\partial t_{2}}|{}_{\left(x_{l},x_{l}\right)},\\ =\theta_{2}\exp\left\{\lambda x_{l}-\frac{\theta_{0}+\theta_{1}+\theta_{2}}{\lambda }\left(e^{\lambda x_{l}}-1\right)\right\}. \end{array}$$

Then, we obtain

(7) $$\begin{array}{c} L\left(x;\lambda ,\theta_{0},\theta_{1},\theta_{2}\right)=\theta_{0}^{n_{0}}\theta_{1}^{n_{1}}\theta_{2}^{n_{2}}\exp\left\{\lambda \sum_{l}x_{l}-\frac{\theta_{0}+\theta_{1}+\theta_{2}}{\lambda }\left[\sum_{l}\left(e^{\lambda x_{l}}-1\right)\right]\right\}. \end{array}$$

## 3. Classical Inference

### 3.1. Maximum Likelihood Estimates (MLEs)

The MLEs of *θ*_0_,  *θ*_1_,  *θ*_2_,  and *λ* can be obtained by maximizing the logarithm of *L*(*x*; *λ*, *θ*_0_, *θ*_1_, *θ*_2_). Set the first partial derivation of log  *L*(*x*; *λ*, *θ*_0_, *θ*_1_, *θ*_2_) about *θ*_0_,  *θ*_1_,  *θ*_2_,  *λ* to 0, i.e.,

(8) $$\begin{array}{c} \frac{\partial \log L\left(x;\lambda ,\theta_{0},\theta_{1},\theta_{2}\right)}{\partial \lambda }=\sum_{l}x_{l}+\frac{\theta_{0}+\theta_{1}+\theta_{2}}{\lambda^{2}}\left[\sum_{l}\left(e^{\lambda x_{l}}-1\right)\right]-\frac{\theta_{0}+\theta_{1}+\theta_{2}}{\lambda }\left(\sum_{l}x_{l}e^{\lambda x_{l}}\right)=0,\\ \frac{\partial \log L\left(x;\lambda ,\theta_{0},\theta_{1},\theta_{2}\right)}{\partial \theta_{j}}=\frac{n_{j}}{\theta_{j}}-\frac{1}{\lambda }\left[\sum_{l}\left(e^{\lambda x_{l}}-1\right)\right]=0,\;j=0,1,2. \end{array}$$

From (8), we get the MLEs of *θ*_0_,  *θ*_1_,  and *θ*_2_ as

(9) $$\begin{array}{c} {\hat{\theta }}_{j}\left(\lambda \right)=\frac{n_{j}\lambda }{\left[\sum_{l}\left(e^{\lambda x_{l}}-1\right)\right]},\;j=0,1,2. \end{array}$$

Substituting ${\hat{\theta }}_{j}\left(\lambda \right)$ into log  *L*(*x*; *λ*, *θ*_0_, *θ*_1_, *θ*_2_), we obtain

(10) $$\begin{array}{c} h\left(\lambda \right)=\lambda \sum_{l}x_{l}+{\sum_{j=0}^{2}\ln\left(\frac{n_{j}\lambda }{\sum_{l}\left(e^{\lambda x_{l}}-1\right)}\right)}^{n_{j}}, \end{array}$$

which is the profile logarithm likelihood function of *λ*.

We can show that *∂*^2^*h*(*λ*)/*∂λ*^2^ < 0, which implies that *h*(*λ*) is concave. Some iterative schemes can be used to find the MLE for *λ*, such as Newton–Raphson algorithm.

### 3.2. Bootstrap Confidence Intervals (CIs)

Since it is hard to construct the exact CIs for the unknown parameters, we consider the Bootstrap method to construct CIs for parameters *θ*_0_,  *θ*_1_,  *θ*_2_,  and *λ*. The Bootstrap method is a resampling method to estimate some statistical characteristics for the unknown parameters by taking samples from the original samples repeatedly; the obtained samples are called Bootstrap samples. This method has a great practical value since it does not need to assume the overall distribution or construct the pivot quantity. We generate the Bootstrap sample by the following three steps:

Step 1: for the fixed value of *n* and observed data (*x*_1_, *x*_2_, ⋯, *x*_*n*_), we get the estimates $\hat{\lambda },\;{\hat{\theta }}_{\text{0}},\;{\hat{\theta }}_{1},\text{ and }{\hat{\theta }}_{2}$ based on the maximum likelihood method.
Step 2: for the values of *n*, $\hat{\lambda },\;{\hat{\theta }}_{\text{0}},\;{\hat{\theta }}_{1},\text{ and }{\hat{\theta }}_{2}$, we generate the sample (*x*_1_^*∗*^, *x*_2_^*∗*^, ⋯, *x*_*n*_^*∗*^). Then, get the MLEs ${\hat{\lambda }}^{'\prime },\;{\hat{\theta }}_{0}^{'\prime },\;{\hat{\theta }}_{1}^{'\prime },\text{ and }{\hat{\theta }}_{2}^{'\prime }$.
Step 3: repeat Step 2 *M* times to obtain *M* sets of the values ${\hat{\lambda }}^{'\prime },\;{\hat{\theta }}_{0}^{'\prime },\;{\hat{\theta }}_{1}^{'\prime },\text{ and }{\hat{\theta }}_{2}^{'\prime }$. Arrange them as follows to get the Bootstrap sample:
  (11) $$\begin{array}{c} \left\{{\hat{\theta }}_{0\left[1\right]}^{'\prime }<\cdots <{\hat{\theta }}_{0\left[M\right]}^{'\prime },\;{\hat{\theta }}_{1\left[1\right]}^{'\prime }<\cdots <{\hat{\theta }}_{1\left[M\right]}^{'\prime },{\hat{\theta }}_{2\left[1\right]}^{'\prime }<\cdots <{\hat{\theta }}_{2\left[M\right]}^{'\prime },{\hat{\lambda }}_{\left[1\right]}^{'\prime }<\cdots <{\hat{\lambda }}_{\left[M\right]}^{'\prime }\right\}. \end{array}$$

Based on the Bootstrap sample and by percentile Bootstrap (Boot-P) method, we construct the Boot-P CIs for *θ*_0_,  *θ*_1_,  *θ*_2_,  *λ* at 1 − *γ* confidence level as

(12) $$\begin{array}{c} \left({\hat{\theta }}_{0\left[M\gamma /2\right]}^{\prime \prime },{\hat{\theta }}_{0\left[M\left(1-\gamma /2\right)\right]}^{\prime \prime }\right),\left({\hat{\theta }}_{1\left[M\gamma /2\right]}^{\prime \prime },{\hat{\theta }}_{1\left[M\left(1-\gamma /2\right)\right]}^{\prime \prime }\right),\left({\hat{\theta }}_{2\left[M\gamma /2\right]}^{\prime \prime },{\hat{\theta }}_{2\left[M\left(1-\gamma /2\right)\right]}^{\prime \prime }\right),\left(\lambda_{\left[M\gamma /2\right]}^{\prime \prime },\lambda_{\left[M\left(1-\gamma /2\right)\right]}^{\prime \prime }\right). \end{array}$$

## 4. Bayesian Inference and HPD CIs

### 4.1. Conjugate Prior

In this section, we suppose the shape parameter *λ* is known. Denote *θ*=*θ*_0_+*θ*_1_+*θ*_2_, which has a Gamma prior with hyperparameters *a* and *b* as

(13) $$\begin{array}{c} \pi \left(\theta \right)=\left(\frac{b^{a}}{\Gamma \left(a\right)}\right)\theta^{a-1}e^{-b\theta },\\ a>0,\;b>0,\;\theta >0. \end{array}$$

Due to *θ*_0_/*θ*+*θ*_1_/*θ*+*θ*_2_/*θ*=1, so given *θ*, (*θ*_1_/*θ*,  *θ*_2_/*θ*) follows a Dirichlet prior with hyper parameters *c*_0_,  *c*_1_, and *c*_2_, that is,

(14) $$\begin{array}{c} \pi_{D}\left(\frac{\theta_{1}}{\theta },\;\frac{\theta_{2}}{\theta }|\theta \right)=\frac{\Gamma \left(\sum_{i=0}^{2}c_{i}\right)}{\prod_{i=0}^{2}\Gamma \left(c_{i}\right)}\prod_{i=0}^{2}{\left(\frac{\theta_{i}}{\theta }\right)}^{c_{i}-1},\;\theta_{i}>0,\;c_{i}>0,\;i=0,\;1,\;2. \end{array}$$

Therefore, the joint prior of *θ*_0_, *θ*_1_,  and *θ*_2_ becomes

(15) $$\begin{array}{c} \pi_{1}\left(\theta_{\text{0}},\;\theta_{1},\;\theta_{2};a,b,c_{0},c_{1},c_{2}\right)=\frac{\Gamma \left(c\right)}{\Gamma \left(a\right)}{\left(b\theta \right)}^{a-c}\prod_{i=0}^{2}\frac{b^{c_{i}}}{\Gamma \left(c_{i}\right)}\theta_{i}^{c_{i}-1}\exp\left(-b\theta_{i}\right), \end{array}$$

where *c*=*c*_0_+*c*_1_+*c*_2_.

### 4.2. Jeffreys Prior

According to Jeffreys [20], Jeffreys prior is proportional to the square root of the determinant of the Fisher information matrix. From (7), we obtain the Fisher information matrix of (*θ*_0_, *θ*_1_,  *θ*_2_) as

(16) $$\begin{array}{c} I=I\left(\theta_{\text{0}}\text{,}\theta_{1},\theta_{2}\right)=\left(\begin{array}{ccc} \frac{n_{0}}{\theta_{0}^{2}} & 0 & 0\\ 0 & \frac{n_{1}}{\theta_{1}^{2}} & 0\\ 0 & 0 & \frac{n_{2}}{\theta_{2}^{2}} \end{array}\right). \end{array}$$

From Theorem 1, we have *n*_*i*_=*n* · *θ*_*i*_/(*θ*_0_+*θ*_1_+*θ*_2_),  *i*=0,  1,  2, so *I*(*θ*_0_, *θ*_1_,  *θ*_2_) can be written as

(17) $$\begin{array}{c} I=n\left(\begin{array}{ccc} \frac{1}{\left(\theta_{0}\theta \right)} & 0 & 0\\ 0 & \frac{1}{\left(\theta_{1}\theta \right)} & 0\\ 0 & 0 & \frac{1}{\left(\theta_{2}\theta \right)} \end{array}\right). \end{array}$$

Thus, the Jeffreys prior is given by

(18) $$\begin{array}{c} \pi_{2}\left(\theta_{\text{0}}\text{, }\theta_{1},\;\theta_{2}\right)\propto \sqrt{\frac{1}{\theta_{\text{0}}\theta_{1}\theta_{2}\theta^{3}}}. \end{array}$$

**Theorem 2 .**

Based on the Jeffreys prior *π*_2_(*θ*_0_, *θ*_1_,  *θ*_2_), the joint posterior distribution of (*θ*_0_, *θ*_1_,  *θ*_2_) is proper.

**Proof**

From (6) and (7), we obtain the joint posterior distribution of (*θ*_0_, *θ*_1_,  *θ*_2_) based on *π*_2_(*θ*_0_, *θ*_1_,  *θ*_2_) as

(19) $$\begin{array}{c} \pi_{2}\left(\theta_{\text{0}}\text{, }\theta_{1},\;\theta_{2}|x\right)=\frac{L\left(x;\lambda ,\theta_{0},\theta_{1},\theta_{2}\right)\pi_{2}\left(\theta_{0},\theta_{1},\theta_{2}\right)}{\int_{0}^{\infty }\int_{0}^{\infty }\int_{0}^{\infty }L\left(x;\lambda ,\theta_{0},\theta_{1},\theta_{2}\right)\pi_{2}\left(\theta_{0},\theta_{1},\theta_{2}\right)d\theta_{0}d\theta_{1}d\theta_{2}},\\ \propto \theta^{\left(-3/2\right)}\theta_{0}^{\left(n_{0}-1/2\right)}\theta_{1}^{\left(n_{1}-1/2\right)}\theta_{2}^{\left(n_{2}-1/2\right)}e^{\left(-A\theta /\lambda \right)}. \end{array}$$

Integrating *π*_2_(*θ*_0_, *θ*_1_,  *θ*_2_*|x*) with respect to *θ*_0_, *θ*_1_,  and *θ*_2_, we obtain

(20) $$\begin{array}{c} \int_{\text{0}}^{\infty }\int_{\text{0}}^{\infty }\int_{\text{0}}^{\infty }\theta^{-3/2}\theta_{0}^{n_{0}-1/2}\theta_{1}^{n_{1}-1/2}\theta_{2}^{n_{2}-1/2}e^{\left(-A\theta /\lambda \right)}d\theta_{0}d\theta_{1}d\theta_{2},\\ =\int_{\text{0}}^{\infty }\int_{\text{0}}^{\infty }\int_{\text{0}}^{\infty }{\left(\frac{\theta_{\text{0}}}{\theta }\right)}^{n_{0}-1/2}{\left(\frac{\theta_{1}}{\theta }\right)}^{n_{1}-1/2}{\left(\frac{\theta_{2}}{\theta }\right)}^{n_{2}-1/2}\theta^{n-3}\exp\left\{-\left(\frac{A}{\lambda }\right)\theta \right\}d\theta_{0}d\theta_{1}d\theta_{2},\\ =\int_{\text{0}<\theta_{\text{0}}/\theta +\theta_{1}/\theta <1}\prod_{i=0}^{1}{\left(\frac{\theta_{i}}{\theta }\right)}^{n_{i}-1/2}{\left(1-\sum_{i=0}^{1}\frac{\theta_{i}}{\theta }\right)}^{n_{2}-1/2}d\frac{\theta_{0}}{\theta }d\frac{\theta_{1}}{\theta }\cdot \int_{0}^{\infty }\theta^{n-1}\exp\left\{-\frac{A}{\lambda }\theta \right\}d\theta ,\\ =B\left(n_{0}+\frac{1}{2},n_{1}+n_{2}+1\right)B\left(n_{1}+\frac{1}{2},n_{2}+\frac{1}{2}\right)\frac{\Gamma \left(n\right)}{{\left(A/\lambda \right)}^{n}}<\infty , \end{array}$$

where *A*=∑_*i*=1_^*n*^(*e*^*λx*_*i*_^ − 1) and *B*(·,  ·) is a beta function.

Thus, the joint posterior distribution of (*θ*_0_, *θ*_1_,  *θ*_2_) based on *π*_2_(*θ*_0_, *θ*_1_,  *θ*_2_) is proper.

### 4.3. Reference Priors

Bernardo [18] and Berger and Bernardo [19] proposed the reference prior which plays a vital role in the objective Bayesian inference. We set *μ*_0_ ≡ *θ*=*θ*_0_+*θ*_1_+*θ*_2_, *μ*_1_=*θ*_0_/*θ*, and *μ*_2_=*θ*_1_/*θ*; the transformation from (*θ*_0_,  *θ*_1_,  *θ*_2_) to (*μ*_0_,  *μ*_1_,  *μ*_2_) is one-to-one with the inverse transformation *θ*_0_=*μ*_0_*μ*_1_, *θ*_1_=*μ*_0_*μ*_2_, and *θ*_2_=*μ*_0_(1 − *μ*_1_ − *μ*_2_). The Jacobian matrix of the transformation has the form

(21) $$\begin{array}{c} J=\left(\begin{array}{ccc} \mu_{1} & \mu_{0} & 0\\ \mu_{2} & 0 & \mu_{0}\\ 1-\mu_{1}-\mu_{2} & -\mu_{0} & -\mu_{0} \end{array}\right),0<\mu_{0}<\infty ,\;0<\mu_{1}+\mu_{2}<1. \end{array}$$

The likelihood function (3) becomes

(22) $$\begin{array}{c} L\left(x;\lambda ,\mu_{0},\mu_{1},\mu_{2}\right)=\mu_{1}^{n_{0}}\mu_{2}^{n_{1}}{\left(1-\mu_{1}-\mu_{2}\right)}^{n_{2}}\mu_{0}^{n}\exp\left\{\lambda \sum_{l}x_{l}-\left(\frac{\mu_{0}}{\lambda }\right)\left[\sum_{l}\left(e^{\lambda x_{l}}-1\right)\right]\right\}. \end{array}$$

The Fisher information matrix of (*μ*_0_,  *μ*_1_,  *μ*_2_) can be written as

(23) $$\begin{array}{c} I_{1}=J^{\prime }IJ=n\left(\begin{array}{ccc} 1/\mu_{0}^{2} & 0 & 0\\ 0 & \frac{1}{\mu_{1}}+\frac{1}{\left(1-\mu_{1}-\mu_{2}\right)} & \frac{1}{\left(1-\mu_{1}-\mu_{2}\right)}\\ 0 & \frac{1}{\left(1-\mu_{1}-\mu_{2}\right)} & \frac{1}{\mu_{2}}+\frac{1}{\left(1-\mu_{1}-\mu_{2}\right)} \end{array}\right). \end{array}$$

**Theorem 3 .**

- Under the ordering groups {*μ*_0_,  (*μ*_1_,  *μ*_2_)} and {(*μ*_1_,  *μ*_2_), *μ*_0_}, the reference priors are the same, which is given by $\omega_{R_{1}}\left(\mu_{\text{0}},\;\mu_{1},\;\mu_{2}\right)=\sqrt{1/\mu_{0}^{2}\mu_{1}\mu_{2}\left(1-\mu_{1}-\mu_{2}\right)}$; the corresponding reference prior for (*θ*_0_, *θ*_1_,  *θ*_2_) is $\pi_{2}\left(\theta_{\text{0}},\;\theta_{1},\;\theta_{2}\right)=\sqrt{1/\theta_{\text{0}}\theta_{1}\theta_{2}\theta^{3}}$
- Under the ordering groups {*μ*_0_,  *μ*_1_,  *μ*_2_}, {*μ*_0_,  *μ*_2_, *μ*_1_}, {*μ*_1_,  *μ*_0_,  *μ*_2_}, and {*μ*_1_,  *μ*_2_,  *μ*_0_}, the reference priors are the same, which is given by $\omega_{R_{2}}\left(\mu_{\text{0}},\;\mu_{1},\;\mu_{2}\right)=\sqrt{1/\mu_{0}^{2}\mu_{1}\mu_{2}\left(1-\mu_{1}-\mu_{2}\right)\left(1-\mu_{1}\right)}$; the corresponding reference prior for (*θ*_0_, *θ*_1_,  *θ*_2_) is $\pi_{3}\left(\theta_{\text{0}},\;\theta_{1},\;\theta_{2}\right)=\sqrt{1/\theta^{2}\theta_{\text{0}}\theta_{1}\theta_{2}\left(\theta_{1}+\theta_{2}\right)}$
- Under the ordering groups {*μ*_2_,  *μ*_0_,  *μ*_1_} and {*μ*_2_,  *μ*_1_, *μ*_0_}, the reference priors are the same, which is given by $\omega_{R_{3}}\left(\mu_{\text{0}},\;\mu_{1},\;\mu_{2}\right)=\sqrt{1/\mu_{0}^{2}\mu_{1}\mu_{2}\left(1-\mu_{1}-\mu_{2}\right)\left(1-\mu_{2}\right)}$; the corresponding reference prior for (*θ*_0_, *θ*_1_,  *θ*_2_) is $\pi_{4}\left(\theta_{\text{0}},\;\theta_{1},\;\left.\theta_{2}\right)\right)=\sqrt{1/\theta^{2}\theta_{\text{0}}\theta_{1}\theta_{2}\left(\theta_{\text{0}}+\theta_{2}\right)}$

**Proof**

(i) The Fisher information matrix of (*μ*_0_,  *μ*_1_,  *μ*_2_) is
  (24) $$\begin{array}{c} I_{1}=\left(\begin{array}{cc} \sum_{11} & \text{0}\\ \text{0} & \sum_{22} \end{array}\right), \end{array}$$
where ∑_11_=n/*μ*_0_^2^ and $\sum_{22}=n\left(\begin{array}{cc} 1/\mu_{1}+1/\left(1-\mu_{1}-\mu_{2}\right) & 1/\left(1-\mu_{1}-\mu_{2}\right)\\ 1/\left(1-\mu_{1}-\mu_{2}\right) & 1/\mu_{2}+1/\left(1-\mu_{1}-\mu_{2}\right) \end{array}\right)$.
The reference prior for the ordering groups {*μ*_0_,  (*μ*_1_,  *μ*_2_)} and {(*μ*_1_,  *μ*_2_), *μ*_0_} is the same as in [21], which is given by
  (25) $$\begin{array}{c} \omega_{R_{1}}\left(\mu_{\text{0}},\;\mu_{1},\;\mu_{2}\right)\propto {\left|\sum_{11}\right|}^{1/2}{\left|\sum_{22}\right|}^{1/2}\propto \sqrt{\frac{1}{\mu_{0}^{2}\mu_{1}\mu_{2}\left(1-\mu_{1}-\mu_{2}\right)}}. \end{array}$$
(ii) The inverse of *I*_1_ is
  (26) $$\begin{array}{c} H=\frac{1}{n}\left(\begin{array}{ccc} \mu_{0}^{2} & 0 & 0\\ 0 & \mu_{1}\left(1-\mu_{1}\right) & -\mu_{1}\mu_{2}\\ 0 & -\mu_{1}\mu_{2} & \mu_{2}\left(1-\mu_{2}\right) \end{array}\right). \end{array}$$
(iii) According the notations in [18], we obtain *h*_1_=1/*μ*_0_^2^, *h*_2_=1/*μ*_1_(1 − *μ*_1_), and *h*_3_=(1 − *μ*_1_)/(*μ*_2_(1 − *μ*_1_ − *μ*_2_)).
Choose the compact sets Ω_*k*_={(*μ*_0_, *μ*_1_, *μ*_2_)*|a*_0*k*_ < *μ*_0_ < *b*_0*k*_,  *a*_1*k*_ < *μ*_1_,  *a*_2*k*_ < *μ*_2_,  *μ*_1_+*μ*_2_ < *d*_*k*_}, such that *a*_0*k*_,  *a*_1*k*_,  *a*_2*k*_⟶0, *b*_0*k*_⟶*∞*, and *d*_*k*_⟶1, as *k*⟶*∞*. Then, we have
  (27) $$\begin{array}{c} \pi^{k}\left(\mu_{0},\;\mu_{1},\;\mu_{2}\right)=\frac{\sqrt{\left|h_{1}\right|}\sqrt{\left|h_{2}\right|}\sqrt{\left|h_{3}\right|}}{\int_{a_{0k}}^{b_{0k}}\sqrt{\left|h_{1}\right|}d\mu_{0}\cdot \int_{a_{1k}}^{d_{k}-\mu_{2}^{\text{0}}}\sqrt{\left|h_{2}\right|}d\mu_{1}\cdot \int_{a_{2k}}^{d_{k}-\mu_{1}}\sqrt{\left|h_{3}\right|}d\mu_{2}}I_{\Omega_{k}}\left(\mu_{0},\mu_{1},\mu_{2}\right), \end{array}$$
where $\int_{a_{0k}}^{b_{0k}}\sqrt{\left|h_{1}\right|}d\mu_{0}=\int_{a_{0k}}^{b_{0k}}1/\mu_{\text{0}}d\mu_{0}=\log\;b_{0k}-\log\;a_{0k}$:
  (28) $$\begin{array}{c} \int_{a_{1k}}^{d_{k}-\mu_{2}^{\text{0}}}\sqrt{\left|h_{2}\right|}d\mu_{1}=\int_{a_{1k}}^{d_{k}-\mu_{2}^{\text{0}}}\sqrt{\frac{1}{\mu_{1}\left(1-\mu_{1}\right)}}d\mu_{1}=-\arcsin\left(1-2\left(d_{k}-\mu_{2}^{\text{0}}\right)\right)+\arcsin\left(1-2a_{1k}\right),\\ \int_{a_{2k}}^{d_{k}-\mu_{1}}\sqrt{\left|h_{3}\right|}d\mu_{2}=\int_{a_{2k}}^{d_{k}-\mu_{1}}\sqrt{\frac{1-\mu_{1}}{\mu_{2}\left(1-\mu_{1}-\mu_{2}\right)}}d\mu_{2},\\ ={\left(1-\mu_{1}\right)}^{1/2}\left(-\arcsin\left(\frac{1-\mu_{1}-2\left(d_{k}-\mu_{1}\right)}{1-\mu_{1}}\right)+\arcsin\left(\frac{1-\mu_{1}-2a_{2k}}{1-\mu_{1}}\right)\right). \end{array}$$
Then, we get the reference prior as
  (29) $$\begin{array}{c} \omega_{R_{2}}\left(\mu_{\text{0}},\;\mu_{1},\;\mu_{2}\right)=\underset{k⟶\infty }{\lim}\frac{\pi^{k}\left(\mu_{0},\;\mu_{1},\;\mu_{2}\right)}{\pi^{k}\left(\mu_{0}^{*},\;\mu_{1}^{*},\;\mu_{2}^{*}\right)}\propto \sqrt{\frac{1}{\mu_{0}^{2}\mu_{1}\mu_{2}\left(1-\mu_{1}-\mu_{2}\right)\left(1-\mu_{1}\right)}}, \end{array}$$
where (*μ*_0_^*∗*^,  *μ*_1_^*∗*^,  *μ*_2_^*∗*^) is an inner point of Ω_*k*_.
Similarly, under the ordering group {*μ*_0_,  *μ*_2_,  *μ*_1_}, the reference prior is *ω*_*R*_2__(*μ*_0_,  *μ*_1_,  *μ*_2_).
The Fisher information matrix of {*μ*_1_,  *μ*_0_,  *μ*_2_} is
  (30) $$\begin{array}{c} I_{2}=n\left(\begin{array}{ccc} \frac{1}{\mu_{1}}+\frac{1}{\left(1-\mu_{1}-\mu_{2}\right)} & 0 & \frac{1}{\left(1-\mu_{1}-\mu_{2}\right)}\\ 0 & \frac{1}{\mu_{0}^{2}} & \text{0}\\ \frac{1}{\left(1-\mu_{1}-\mu_{2}\right)} & \text{0} & \frac{1}{\mu_{2}}+\frac{1}{\left(1-\mu_{1}-\mu_{2}\right)} \end{array}\right). \end{array}$$
The inverse of *I*_2_ is
  (31) $$\begin{array}{c} H_{1}=\frac{1}{n}\left(\begin{array}{ccc} \mu_{1}\left(1-\mu_{1}\right) & 0 & -\mu_{1}\mu_{2}\\ 0 & \mu_{0}^{2} & 0\\ -\mu_{1}\mu_{2} & 0 & \mu_{2}\left(1-\mu_{2}\right) \end{array}\right). \end{array}$$
Similarly, we obtain *h*_1_=1/*μ*_1_(1 − *μ*_1_), *h*_2_=1/*μ*_0_^2^, and *h*_3_=(1 − *μ*_1_)/(*μ*_2_(1 − *μ*_1_ − *μ*_2_)).
Choose the compact sets Ω_*k*_={(*μ*_1_, *μ*_0_, *μ*_2_)*|a*_0*k*_ < *μ*_1_,  *a*_1*k*_ < *μ*_0_ < *b*_1*k*_,  *a*_2*k*_ < *μ*_2_,  *μ*_1_+*μ*_2_ < *d*_*k*_}, such that *a*_0*k*_,  *a*_1*k*_,  *a*_2*k*_⟶0, *b*_1*k*_⟶*∞*, and *d*_*k*_⟶1, as *k*⟶*∞*. Then, we have
  (32) $$\begin{array}{c} \pi^{k}\left(\mu_{1},\;\mu_{\text{0}},\;\mu_{2}\right)=\frac{\sqrt{\left|h_{1}\right|}\sqrt{\left|h_{2}\right|}\sqrt{\left|h_{3}\right|}}{\int_{a_{0k}}^{d_{k}-u_{2}^{\text{0}}}\sqrt{\left|h_{1}\right|}d\mu_{1}\cdot \int_{a_{1k}}^{b_{1k}}\sqrt{\left|h_{2}\right|}d\mu_{\text{0}}\cdot \int_{a_{2k}}^{d_{k}-\mu_{1}}\sqrt{\left|h_{3}\right|}d\mu_{2}}I_{\Omega_{k}}\left(\mu_{1},\mu_{0},\mu_{2}\right), \end{array}$$
where $\int_{a_{0k}}^{d_{k}-u_{2}^{\text{0}}}\sqrt{\left|h_{1}\right|}d\mu_{1}=\int_{a_{0k}}^{d_{k}-u_{2}^{\text{0}}}\sqrt{1/\mu_{1}\left(1-\mu_{1}\right)}d\mu_{1}=-\arcsin\left(1-2\left(d_{k}-u_{2}^{\text{0}}\right)\right)+\arcsin\left(1-2a_{0k}\right)$,
  (33) $$\begin{array}{c} \int_{a_{1k}}^{b_{1k}}\sqrt{\left|h_{2}\right|}d\mu_{\text{0}}=\int_{a_{1k}}^{b_{1k}}\frac{1}{\mu_{\text{0}}}d\mu_{\text{0}}=\log\;b_{1k}-\log\;a_{1k},\\ \int_{a_{2k}}^{d_{k}-\mu_{1}}\sqrt{\left|h_{3}\right|}d\mu_{2}=\int_{a_{2k}}^{d_{k}-\mu_{1}}\sqrt{\frac{1-\mu_{1}}{\mu_{2}\left(1-\mu_{1}-\mu_{2}\right)}}d\mu_{2},\\ ={\left(1-\mu_{1}\right)}^{1/2}\left(-\arcsin\left(\frac{1-\mu_{1}-2\left(d_{k}-u_{1}\right)}{1-\mu_{1}}\right)+\arcsin\left(\frac{1-\mu_{1}-2a_{2k}}{1-\mu_{1}}\right)\right). \end{array}$$
Let (*μ*_1_^*∗*^,  *μ*_0_^*∗*^,  *μ*_2_^*∗*^) be an inner point of Ω_*k*_; we get the reference prior as
  (34) $$\begin{array}{c} \omega_{R_{2}}\left(\mu_{\text{0}},\;\mu_{1},\;\mu_{2}\right)=\underset{k⟶\infty }{\lim}\frac{\pi^{k}\left(\mu_{1},\;\mu_{\text{0}},\;\mu_{2}\right)}{\pi^{k}\left(\mu_{1}^{*},\;\mu_{\text{0}}^{*},\;\mu_{2}^{*}\right)}\propto \sqrt{\frac{1}{\mu_{0}^{2}\mu_{1}\mu_{2}\left(1-\mu_{1}-\mu_{2}\right)\left(1-\mu_{1}\right)}}. \end{array}$$
Similarly, under the ordering group {*μ*_1_,  *μ*_2_,  *μ*_0_}, the reference prior is *ω*_*R*_2__(*μ*_0_,  *μ*_1_,  *μ*_2_).
(v) The Fisher information matrix of {*μ*_2_,  *μ*_1_,  *μ*_0_} is

(35) $$\begin{array}{c} I_{3}=n\left(\begin{array}{ccc} \frac{1}{\mu_{2}}+\frac{1}{\left(1-\mu_{1}-\mu_{2}\right)} & \frac{1}{\left(1-\mu_{1}-\mu_{2}\right)} & 0\\ \frac{1}{\left(1-\mu_{1}-\mu_{2}\right)} & \frac{1}{\mu_{1}}+\frac{1}{\left(1-\mu_{1}-\mu_{2}\right)} & \text{0}\\ 0 & \text{0} & \frac{1}{\mu_{0}^{2}} \end{array}\right). \end{array}$$

The inverse of *I*_3_ is

(36) $$\begin{array}{c} H_{2}=\frac{1}{n}\left(\begin{array}{ccc} \mu_{2}\left(1-\mu_{2}\right) & -\mu_{1}\mu_{2} & 0\\ -\mu_{1}\mu_{2} & \mu_{1}\left(1-\mu_{1}\right) & 0\\ 0 & 0 & \mu_{0}^{2} \end{array}\right). \end{array}$$

Then, we obtain *h*_1_=1/*μ*_2_(1 − *μ*_2_), *h*_2_=(1 − *μ*_2_)/(*μ*_1_(1 − *μ*_1_ − *μ*_2_)), and *h*_3_=1/*μ*_0_^2^.

Choose the compact sets Ω_*k*_={(*μ*_2_, *μ*_1_, *μ*_0_)*|a*_0*k*_ < *μ*_2_,  *a*_1*k*_ < *μ*_1_,  *μ*_2_+*μ*_1_ < *d*_*k*_, *a*_2*k*_ < *μ*_0_ < *b*_2*k*_}, such that *a*_0*k*_,  *a*_1*k*_,  *a*_2*k*_⟶0, *b*_2*k*_⟶*∞*, and *d*_*k*_⟶1, as *k*⟶*∞*. Then, we have

(37) $$\begin{array}{c} \pi^{k}\left(\mu_{2},\;\mu_{1},\;\mu_{\text{0}}\right)=\frac{\sqrt{\left|h_{1}\right|}\sqrt{\left|h_{2}\right|}\sqrt{\left|h_{3}\right|}}{\int_{a_{0k}}^{d_{k}-u_{1}^{0}}\sqrt{\left|h_{1}\right|}d\mu_{2}\cdot \int_{a_{1k}}^{d_{k}-\mu_{2}}\sqrt{\left|h_{2}\right|}d\mu_{1}\cdot \int_{a_{2k}}^{b_{2k}}\sqrt{\left|h_{3}\right|}d\mu_{\text{0}}}I_{\Omega_{k}}\left(\mu_{2},\mu_{1},\mu_{\text{0}}\right), \end{array}$$

where $\int_{a_{0k}}^{d_{k}-u_{1}^{0}}\sqrt{\left|h_{1}\right|}d\mu_{2}=\int_{a_{0k}}^{d_{k}-u_{1}^{0}}\sqrt{1/\mu_{2}\left(1-\mu_{2}\right)}d\mu_{2}=-\arcsin\left(1-2\left(d_{k}-u_{1}^{0}\right)\right)+\arcsin\left(1-2a_{0k}\right)$,

(38) $$\begin{array}{c} \int_{a_{1k}}^{d_{k}-\mu_{2}}\sqrt{\left|h_{2}\right|}d\mu_{1}=\int_{a_{1k}}^{d_{k}-\mu_{2}}\sqrt{\frac{1-\mu_{2}}{\mu_{1}\left(1-\mu_{1}-\mu_{2}\right)}}d\mu_{1},\\ ={\left(1-\mu_{2}\right)}^{1/2}\left(-\arcsin\left(\frac{1-\mu_{2}-2\left(d_{k}-u_{2}\right)}{1-\mu_{2}}\right)+\arcsin\left(\frac{1-\mu_{2}-2a_{1k}}{1-\mu_{2}}\right)\right),\\ \int_{a_{2k}}^{b_{2k}}\sqrt{\left|h_{3}\right|}d\mu_{0}=\int_{a_{2k}}^{b_{2k}}\frac{1}{\mu_{0}}d\mu_{0}=\log\;b_{2k}-\log\;a_{2k}. \end{array}$$

Let (*μ*_2_^*∗*^,  *μ*_1_^*∗*^,  *μ*_0_^*∗*^) be an inner point of Ω_*k*_, we obtain the reference prior as

(39) $$\begin{array}{c} \omega_{R_{3}}\left(\mu_{\text{0}},\;\mu_{1},\;\mu_{2}\right)=\underset{k⟶\infty }{\lim}\frac{\pi^{k}\left(\mu_{2},\;\mu_{1},\;\mu_{0}\right)}{\pi^{k}\left(\mu_{2}^{*},\;\mu_{1}^{*},\;\mu_{0}^{*}\right)}\propto \sqrt{\frac{1}{\mu_{0}^{2}\mu_{1}\mu_{2}\left(1-\mu_{1}-\mu_{2}\right)\left(1-\mu_{2}\right)}}. \end{array}$$

Similarly, under the ordering group {*μ*_2_,  *μ*_0_,  *μ*_1_}, the reference prior is *ω*_*R*_3__(*μ*_0_,  *μ*_1_,  *μ*_2_). According to the one-to-one transformation from (*μ*_0_,  *μ*_1_,  *μ*_2_) to (*θ*_0_,  *θ*_1_,  *θ*_2_), we can obtain the reference priors *π*_2_(*μ*_0_,  *μ*_1_,  *μ*_2_), *π*_3_(*μ*_0_,  *μ*_1_,  *μ*_2_), *π*_4_(*μ*_0_,  *μ*_1_,  *μ*_2_) from *ω*_*R*_1__, *ω*_*R*_2__, and *ω*_*R*_3__, respectively.

**Theorem 4 .**

Based on the reference priors *π*_3_(*θ*_0_, *θ*_1_,  *θ*_2_) and *π*_4_(*θ*_0_, *θ*_1_,  *θ*_2_), the posterior distributions of (*θ*_0_, *θ*_1_,  *θ*_2_) are proper.

**Proof**

The joint posterior distributions of (*θ*_0_, *θ*_1_,  *θ*_2_) based on reference prior *π*_3_(*θ*_0_, *θ*_1_,  *θ*_2_) and *π*_4_(*θ*_0_, *θ*_1_,  *θ*_2_) are, respectively, as

(40) $$\begin{array}{c} \pi_{3}\left(\theta_{\text{0}}\text{,}\theta_{1},\theta_{2}|x\right)=\frac{L\left(x;\lambda ,\theta_{0},\theta_{1},\theta_{2}\right)\pi_{3}\left(\theta_{0},\theta_{1},\theta_{2}\right)}{\int_{0}^{\infty }\int_{0}^{\infty }\int_{0}^{\infty }L\left(x;\lambda ,\theta_{0},\theta_{1},\theta_{2}\right)\pi_{3}\left(\theta_{0},\theta_{1},\theta_{2}\right)d\theta_{0}d\theta_{1}d\theta_{2}},\\ \propto \theta^{-1}\theta_{0}^{n_{0}-1/2}\theta_{1}^{n_{1}-1/2}\theta_{2}^{n_{2}-1/2}{\left(\theta_{1}+\theta_{2}\right)}^{-1/2}\exp\left\{-\frac{A\theta }{\lambda }\right\},\\ \pi_{4}\left(\theta_{\text{0}}\text{,}\theta_{1},\theta_{2}|x\right)=\frac{L\left(x;\lambda ,\theta_{0},\theta_{1},\theta_{2}\right)\pi_{4}\left(\theta_{0},\theta_{1},\theta_{2}\right)}{\int_{0}^{\infty }\int_{0}^{\infty }\int_{0}^{\infty }L\left(x;\lambda ,\theta_{0},\theta_{1},\theta_{2}\right)\pi_{4}\left(\theta_{0},\theta_{1},\theta_{2}\right)d\theta_{0}d\theta_{1}d\theta_{2}},\\ \propto \theta^{-1}\theta_{0}^{n_{0}-1/2}\theta_{1}^{n_{1}-1/2}\theta_{2}^{n_{2}-1/2}{\left(\theta_{\text{0}}+\theta_{2}\right)}^{-1/2}\exp\left\{-\frac{A\theta }{\lambda }\right\}. \end{array}$$

Integrating *π*_3_(*θ*_0_, *θ*_1_,  *θ*_2_*|x*) and *π*_4_(*θ*_0_, *θ*_1_,  *θ*_2_*|x*) with respect to *θ*_0_, *θ*_1_,  and *θ*_2_, respectively, we obtain

(41) $$\begin{array}{c} \int_{\text{0}}^{\infty }\int_{\text{0}}^{\infty }\int_{\text{0}}^{\infty }\theta^{-1}\theta_{0}^{n_{0}-1/2}\theta_{1}^{n_{1}-1/2}\theta_{2}^{n_{2}-1/2}{\left(\theta_{1}+\theta_{2}\right)}^{-1/2}\exp\left\{-\frac{A\theta }{\lambda }\right\}d\theta_{0}d\theta_{1}d\theta_{2},\\ =B\left(n_{0}+\frac{1}{2},n_{1}+n_{2}+\frac{1}{2}\right)B\left(n_{1}+\frac{1}{2},n_{2}+\frac{1}{2}\right)\frac{\Gamma \left(n\right)}{{\left(A/\lambda \right)}^{n}}<\infty ,\\ \int_{0}^{\infty }\int_{0}^{\infty }\int_{0}^{\infty }\theta^{-1}\theta_{0}^{n_{0}-1/2}\theta_{1}^{n_{1}-1/2}\theta_{2}^{n_{2}-1/2}{\left(\theta_{\text{0}}+\theta_{2}\right)}^{-1/2}\exp\left\{-\frac{A\theta }{\lambda }\right\}d\theta_{0}d\theta_{1}d\theta_{2},\\ =B\left(n_{1}+\frac{1}{2},n_{\text{0}}+n_{2}+\frac{1}{2}\right)B\left(n_{\text{0}}+\frac{1}{2},n_{2}+\frac{1}{2}\right)\frac{\Gamma \left(n\right)}{{\left(A/\lambda \right)}^{n}}<\infty . \end{array}$$

Thus, the posterior distributions of (*θ*_0_, *θ*_1_,  *θ*_2_) based on *π*_3_(*θ*_0_, *θ*_1_,  *θ*_2_) and *π*_4_(*θ*_0_, *θ*_1_,  *θ*_2_) are proper.

### 4.4. Bayesian Estimates

The joint posterior distributions of (*θ*_0_, *θ*_1_,  *θ*_2_) based on *π*_1_,  *π*_2_,  *π*_3_,  and *π*_4_ are, respectively, as

(42) $$\begin{array}{c} \pi_{1}\left(\theta_{\text{0}}\text{, }\theta_{1},\;\theta_{2}|x\right)=\frac{L\left(x;\lambda ,\theta_{0},\theta_{1},\theta_{2}\right)\pi_{1}\left(\theta_{0},\theta_{1},\theta_{2}\right)}{\int_{0}^{\infty }\int_{0}^{\infty }\int_{0}^{\infty }L\left(x;\lambda ,\theta_{0},\theta_{1},\theta_{2}\right)\pi_{1}\left(\theta_{0},\theta_{1},\theta_{2}\right)d\theta_{0}d\theta_{1}d\theta_{2}}, \end{array}$$

where

(43) $$\begin{array}{c} \int_{0}^{\infty }\int_{0}^{\infty }\int_{0}^{\infty }L\left(x;\lambda ,\theta_{0},\theta_{1},\theta_{2}\right)\pi_{1}\left(\theta_{0},\theta_{1},\theta_{2}\right)d\theta_{0}d\theta_{1}d\theta_{2}\\ =w_{1}w_{2}\exp\left\{\lambda \sum_{l}x_{l}\right\}\int_{\text{0}<\theta_{\text{0}}/\theta +\theta_{1}/\theta <1}\prod_{i=0}^{1}{\left(\frac{\theta_{i}}{\theta }\right)}^{n_{i}+c_{i}-1}{\left(1-\sum_{i=0}^{1}\frac{\theta_{i}}{\theta }\right)}^{n_{2}+c_{2}-1}d\frac{\theta_{0}}{\theta }d\frac{\theta_{1}}{\theta },\int_{0}^{\infty }\theta^{n+a+1}e^{-\left(A/\lambda +b\right)\theta }d\theta \\ =w_{1}w_{2}\exp\left\{\lambda \sum_{l}x_{l}\right\}B\left(n_{0}+c_{0},n_{1}+c_{1}+n_{2}+c_{2}\right)B\left(n_{1}+c_{1},n_{2}+c_{2}\right)\frac{\Gamma \left(n+a+2\right)}{{\left(A/\lambda +b\right)}^{n+a+2}}, \end{array}$$

where *w*_1_=Γ(∑_*i*=0_^2^*c*_*i*_)*b*^*a*−*c*_0_−*c*_1_−*c*_2_^/Γ(*a*) and *w*_2_=∏_*i*=0_^2^*b*^*c*_*i*_^/Γ(*c*_*i*_).

Thus, we obtain

(44) $$\begin{array}{c} \pi_{1}\left(\theta_{\text{0}}\text{, }\theta_{1},\;\theta_{2}|x\right)=\frac{\theta^{a-c_{0}-c_{1}-c_{2}}\theta_{0}^{n_{0}+c_{0}-1}\theta_{1}^{n_{1}+c_{1}-1}\theta_{2}^{n_{2}+c_{2}-1}\exp\left\{-\left(A/\lambda +b\right)\theta \right\}}{B\left(n_{0}+c_{0},n_{1}+c_{1}+n_{2}+c_{2}\right)B\left(n_{1}+c_{1},n_{2}+c_{2}\right)\Gamma \left(n+a\right)/{\left(A/\lambda +b\right)}^{n+a}}. \end{array}$$

Similarly,

(45) $$\begin{array}{c} \pi_{2}\left(\theta_{\text{0}}\text{, }\theta_{1},\;\theta_{2}|x\right)=\frac{L\left(x;\lambda ,\theta_{0},\theta_{1},\theta_{2}\right)\pi_{2}\left(\theta_{0},\theta_{1},\theta_{2}\right)}{\int_{0}^{\infty }\int_{0}^{\infty }\int_{0}^{\infty }L\left(x;\lambda ,\theta_{0},\theta_{1},\theta_{2}\right)\pi_{2}\left(\theta_{0},\theta_{1},\theta_{2}\right)d\theta_{0}d\theta_{1}d\theta_{2}}, \end{array}$$

where

(46) $$\begin{array}{c} \int_{0}^{\infty }\int_{0}^{\infty }\int_{0}^{\infty }L\left(x;\lambda ,\theta_{0},\theta_{1},\theta_{2}\right)\pi_{2}\left(\theta_{0},\theta_{1},\theta_{2}\right)d\theta_{0}d\theta_{1}d\theta_{2},\\ =\int_{0}^{\infty }\int_{0}^{\infty }\int_{0}^{\infty }\theta^{-3/2}\theta_{0}^{n_{0}-1/2}\theta_{1}^{n_{1}-1/2}\theta_{2}^{n_{2}-1/2}\exp\left\{\lambda \sum_{l}x_{l}-\frac{A}{\lambda }\theta \right\}d\theta_{0}d\theta_{1}d\theta_{2},\\ =\exp\left\{\lambda \sum_{l}x_{l}\right\}B\left(n_{0}+\frac{1}{2},n_{1}+n_{2}+1\right)B\left(n_{1}+\frac{1}{2},n_{2}+\frac{1}{2}\right)\frac{\Gamma \left(n\right)}{{\left(A/\lambda \right)}^{n}}. \end{array}$$

We obtain

(47) $$\begin{array}{c} \pi_{2}\left(\theta_{\text{0}}\text{, }\theta_{1},\;\theta_{2}|x\right)=\frac{\theta^{-3/2}\theta_{0}^{n_{0}-1/2}\theta_{1}^{n_{1}-1/2}\theta_{2}^{n_{2}-1/2}\exp\left\{-A\theta /\lambda \right\}}{B\left(n_{0}+1/2,n_{1}+n_{2}+1\right)B\left(n_{1}+1/2,n_{2}+1/2\right)\Gamma \left(n\right)/{\left(A/\lambda \right)}^{n}},\\ \pi_{3}\left(\theta_{\text{0}}\text{, }\theta_{1},\;\theta_{2}|x\right)=\frac{L\left(x;\lambda ,\theta_{0},\theta_{1},\theta_{2}\right)\pi_{3}\left(\theta_{0},\theta_{1},\theta_{2}\right)}{\int_{0}^{\infty }\int_{0}^{\infty }\int_{0}^{\infty }L\left(x;\lambda ,\theta_{0},\theta_{1},\theta_{2}\right)\pi_{3}\left(\theta_{0},\theta_{1},\theta_{2}\right)d\theta_{0}d\theta_{1}d\theta_{2}}, \end{array}$$

where

(48) $$\begin{array}{c} \int_{0}^{\infty }\int_{0}^{\infty }\int_{0}^{\infty }L\left(x;\lambda ,\theta_{0},\theta_{1},\theta_{2}\right)\pi_{3}\left(\theta_{0},\theta_{1},\theta_{2}\right)d\theta_{0}d\theta_{1}d\theta_{2},\\ =\int_{0}^{\infty }\int_{0}^{\infty }\int_{0}^{\infty }\theta^{-1}\theta_{0}^{n_{0}-1/2}\theta_{1}^{n_{1}-1/2}\theta_{2}^{n_{2}-1/2}{\left(\theta_{1}+\theta_{2}\right)}^{-1/2}\exp\left\{\lambda \sum_{l}x_{l}-\frac{A\theta }{\lambda }\right\}d\theta_{0}d\theta_{1}d\theta_{2},\\ =\exp\left\{\lambda \sum_{l}x_{l}\right\}B\left(n_{0}+\frac{1}{2},n_{1}+n_{2}+\frac{1}{2}\right)B\left(n_{1}+\frac{1}{2},n_{2}+\frac{1}{2}\right)\frac{\Gamma \left(n\right)}{{\left(A/\lambda \right)}^{n}}. \end{array}$$

We obtain

(49) $$\begin{array}{c} \pi_{3}\left(\theta_{\text{0}}\text{, }\theta_{1},\;\theta_{2}|x\right)=\frac{\theta^{-1}\theta_{0}^{n_{0}-1/2}\theta_{1}^{n_{1}-1/2}\theta_{2}^{n_{2}-1/2}{\left(\theta_{1}+\theta_{2}\right)}^{-1/2}\exp\left\{-A\theta /\lambda \right\}}{B\left(n_{0}+1/2,n_{1}+n_{2}+1/2\right)B\left(n_{1}+1/2,n_{2}+1/2\right)\Gamma \left(n\right)/{\left(A/\lambda \right)}^{n}},\\ \pi_{4}\left(\theta_{\text{0}}\text{, }\theta_{1},\;\theta_{2}|x\right)=\frac{L\left(x;\lambda ,\theta_{0},\theta_{1},\theta_{2}\right)\pi_{4}\left(\theta_{0},\theta_{1},\theta_{2}\right)}{\int_{0}^{\infty }\int_{0}^{\infty }\int_{0}^{\infty }L\left(x;\lambda ,\theta_{0},\theta_{1},\theta_{2}\right)\pi_{4}\left(\theta_{0},\theta_{1},\theta_{2}\right)d\theta_{0}d\theta_{1}d\theta_{2}}, \end{array}$$

where

(50) $$\begin{array}{c} \int_{0}^{\infty }\int_{0}^{\infty }\int_{0}^{\infty }L\left(x;\lambda ,\theta_{0},\theta_{1},\theta_{2}\right)\pi_{4}\left(\theta_{0},\theta_{1},\theta_{2}\right)d\theta_{0}d\theta_{1}d\theta_{2},\\ =\int_{0}^{\infty }\int_{0}^{\infty }\int_{0}^{\infty }\theta^{-1}\theta_{0}^{n_{0}-1/2}\theta_{1}^{n_{1}-1/2}\theta_{2}^{n_{2}-1/2}{\left(\theta_{\text{0}}+\theta_{2}\right)}^{-1/2}\exp\left\{\lambda \sum_{l}x_{l}-\frac{A\theta }{\lambda }\right\}d\theta_{0}d\theta_{1}d\theta_{2},\\ =\exp\left\{\lambda \sum_{l}x_{l}\right\}B\left(n_{1}+\frac{1}{2},n_{\text{0}}+n_{2}+\frac{1}{2}\right)B\left(n_{\text{0}}+\frac{1}{2},n_{2}+\frac{1}{2}\right)\frac{\Gamma \left(n\right)}{{\left(A/\lambda \right)}^{n}}. \end{array}$$

Then, we have

(51) $$\begin{array}{c} \pi_{4}\left(\theta_{\text{0}}\text{, }\theta_{1},\;\theta_{2}|x\right)=\frac{\theta^{-1}\theta_{0}^{n_{0}-1/2}\theta_{1}^{n_{1}-1/2}\theta_{2}^{n_{2}-1/2}{\left(\theta_{\text{0}}+\theta_{2}\right)}^{-1/2}\exp\left\{-A\theta /\lambda \right\}}{B\left(n_{1}+1/2,n_{\text{0}}+n_{2}+1/2\right)B\left(n_{\text{0}}+1/2,n_{2}+1/2\right)\Gamma \left(n\right)/{\left(A/\lambda \right)}^{n}}. \end{array}$$

From (9)–(12), we get the Bayesian estimates of parameters *θ*_0_,  *θ*_1_,  *θ*_2_,  and *θ* against squared error loss function based on *π*_1_,  *π*_2_,  *π*_3_,  and *π*_4_, respectively, which are listed in Table 1.

### 4.5. HPD Credible Intervals

The HPD credible intervals of parameters *θ*_0_,  *θ*_1_,  *θ*_2_,  and *θ* can be constructed by the Monte Carlo method studied by Chen and Shao [22].

Step 1: given the value of *n* and the observed data (*x*_1_, *x*_2_, ⋯, *x*_*n*_), compute the Bayesian estimates of ${\hat{\theta }}_{\text{0}},\;{\hat{\theta }}_{1},\;{\hat{\theta }}_{2},\text{ and }\hat{\theta }$ based on *π*_1_,  *π*_2_,  *π*_3_,  and *π*_4_, respectively.
Step 2: repeat Step 1 *M* times; we obtain *M* sets of the values ${\hat{\theta }}_{\text{0}},\;{\hat{\theta }}_{1},\;{\hat{\theta }}_{2},\text{ and }\hat{\theta }$ based on *π*_1_,  *π*_2_,  *π*_3_,  and *π*_4_, respectively. Arrange them in the ascending order, we obtain
  (52) $$\begin{array}{c} {\hat{\theta }}_{0\pi_{k}\left[1\right]}<\cdots <{\hat{\theta }}_{0\pi_{k}\left[M\right]},{\hat{\theta }}_{1\pi_{k}\left[1\right]}<\cdots <{\hat{\theta }}_{1\pi_{k}\left[M\right]},{\hat{\theta }}_{2\pi_{k}\left[1\right]}<\cdots <{\hat{\theta }}_{2\pi_{k}\left[M\right]},{\hat{\theta }}_{\pi_{k}\left[1\right]}<\cdots <{\hat{\theta }}_{\pi_{k}\left[M\right]},\;k=1,2,3,4. \end{array}$$
Step 3: compute the CIs at 1 − *γ* confidence level as
  (53) $$\begin{array}{c} \left({\hat{\theta }}_{v\pi_{k}\left[w\right]},\;{\hat{\theta }}_{v\pi_{k}\left[w+\left(1-\gamma \right)M\right]}\right),\;\left({\hat{\theta }}_{\pi_{k}\left[w\right]},\;{\hat{\theta }}_{\pi_{k}\left[w+\left(1-\gamma \right)M\right]}\right),v=0,1,2;w=1,2,\cdots ,M-\left(1-\gamma \right)M;k=1,2,3,4. \end{array}$$
  Step 4 : the HPD CIs for *θ*_*v*_, *v*=0,1,2, and *θ* are the shortest intervals among $\left({\hat{\theta }}_{v\pi_{k}\left[w\right]},\;{\hat{\theta }}_{v\pi_{k}\left[w+\left(1-\gamma \right)M\right]}\right),\left({\hat{\theta }}_{\pi_{k}\left[w\right]},\;{\hat{\theta }}_{\pi_{k}\left[w+\left(1-\gamma \right)M\right]}\right),$ and *w*=1,  2, ⋯, *M* − (1 − *γ*)*M*, respectively.

## 5. Numerical Simulation and Illustrative Example

### 5.1. Simulation

Suppose the common shape parameter *λ* is known. The initial values for parameters (*λ*,  *θ*_0_, *θ*_1_,  *θ*_2_) are (3,  1,  2,  1). The initial values for the hyperparameters *a*,  *b*,  *c*_0_,  *c*_1_,  and *c*_2_ are all 0.001. Take the sample size *n* = 10, 20, 30, and 50. Generate the random samples (*x*_1_, *x*_2_, ⋯, *x*_*n*_) from MOGP(*λ*, *θ*_0_, *θ*_1_, *θ*_2_) by the following steps:

  Step 1: for a fixed value *n*, generate *n* samples *u*_01_, *u*_02_, ⋯, *u*_0*n*_ from *GP*(*λ*, *θ*_0_), *u*_11_, *u*_12_, ⋯, *u*_1*n*_ from *GP*(*λ*, *θ*_1_), and *u*_21_, *u*_22_, ⋯, *u*_2*n*_ from *GP*(*λ*, *θ*_2_). Then, we obtain *T*_1*l*_=min(*u*_0*l*_, *u*_1*l*_) and *T*_2*l*_=min(*u*_0*l*_, *u*_2*l*_),  *l*=1,2, ⋯, *n*.
  Step 2: compute (*x*_*l*_, *δ*_0*l*_, *δ*_1*l*_, *δ*_2*l*_),  *l*=1,2, ⋯, *n*, where *x*_*l*_=min(*T*_1*l*_, *T*_2*l*_), *δ*_0*l*_=*I*(*T*_1*l*_=*T*_2*l*_), *δ*_1*l*_=*I*(*T*_1*l*_ < *T*_2*l*_), and *δ*_2*l*_=*I*(*T*_1*l*_ > *T*_2*l*_).

Repeat the procedures 10,000 times; we get the values of the mean squared errors (MSEs) of the MLEs, the average lengths (ALs), and coverage probabilities (CPs) of the 95% Boot-P CIs, and the MSEs of the Bayesian estimates, the ALs, and CPs of the 95% HPD CIs, which are shown in Table 2–5. From the results in Table 2–5, we can make the following conclusions.

The MSEs of MLEs and Bayesian estimates decrease as the sample size increases. For given sample size *n*, the Bayesian estimates based on *π*_1_, *π*_2_,  and *π*_4_ are smaller than the MSEs of MLEs. The MSEs of Bayesian estimates of *θ*_0_ and *θ*_2_ based on *π*_4_ are smaller than that based on *π*_1_, *π*_2_,  and *π*_3_. The MSEs of Bayesian estimates of *θ*_1_ based on *π*_3_ are smaller than that based on *π*_1_, *π*_2_,  and *π*_4_. The MSEs of Bayesian estimates of *θ* based on *π*_1_ are smaller than that based on *π*_2_, *π*_3_,  and *π*_4_.

The CPs of Boot-P and HPD CIs are all close to 0.95. The ALs of Boot-P and HPD CIs decrease; the associated CPs increase when the sample size increases. The CPs of HPD CIs based on Bayesian estimates are larger than the CPs of Boot-P CIs based on MLEs.

### 5.2. Illustrative Analysis

#### 5.2.1. Simulated Data

For illustrative purposes, with initial value for parameters (*λ*, *θ*_0_, *θ*_1_, *θ*_2_) as (3,1,2,1), we use the procedures mentioned above to generate *U*_0_,  *U*_1_,  and *U*_2_ from *GP*(3,1), *GP*(3,2), and *GP*(3,1), respectively. We then get *T*_1_=min(*U*_0_, *U*_1_) and *T*_2_=min(*U*_0_, *U*_2_); the latent lifetime of the system is min(*T*_1_, *T*_2_). The simulated data are listed in Table 6. The MLEs, Bayesian estimates, and associated 95% CIs for parameters *θ*_0_,  *θ*_1_,  *θ*_2_,  and *θ* are shown in Table 7. From Table 7, all the MLEs and Bayesian estimates of (*θ*_0_,  *θ*_1_,  *θ*_2_, *θ*) are close to the true value.

#### 5.2.2. Real Data

Use the procedures mentioned above to a real dataset. Kundu and Gupta [13] analyzed the football data of UEFA Champions' League data which are presented in Table 1. From the data, *T*_1_ and *T*_2_ can be regarded as two dependent failure modes, and *n*_0_=7, *n*_1_=17,  and *n*_2_=13. This data have been fitted by Marshall–Olkin bivariate Gompertz distribution (see Wang et al. [23]).

The MLEs, Bayesian estimates, and associated 95% CIs for parameters *θ*_0_,  *θ*_1_,  *θ*_2_,  and *θ* are shown in Table 8. From Tables 7 and 8, Bayesian estimates under different priors are close to MLEs, and the lengths of 95% Boot-p CIs associated to MLEs are longer than the lengths of 95% HPD CIs associated to Bayesian estimates.

## 6. Conclusion

This paper discussed the point estimates and CIs for the parameters of the dependent competing risks' model from MOGP distribution. We studied the appropriateness of the posteriors based on conjugate prior and Jeffreys and Reference priors, obtained the Bayesian estimates in closed forms, and constructed the associated HPD CIs. From the simulations results, the use of the Bayesian method can be recommended if the priors are available. The results of the illustrative analysis show that the proposed methods work well; from the lengths of CIs, we can conclude the Bayesian estimates are better than MLEs in general.

## Figures and Tables

**Table 1:** Bayesian estimates of parameters based on different priors.

Prior	*θ*_0_	*θ*_1_	*θ*_2_	*θ*
*π*_*1*_	*λ*(*n*_0_+*c*_0_)(*n*+*a*)/(*n*+*c*_0_+*c*_1_+*c*_2_)(*A*+*bλ*)	*λ*(*n*_1_+*c*_1_)(*n*+*a*)/(*n*+*c*_0_+*c*_1_+*c*_2_)(*A*+*bλ*)	*λ*(*n*_2_+*c*_2_)(*n*+*a*)/(*n*+*c*_0_+*c*_1_+*c*_2_)(*bλ*+*A*)	(*n*+*a*)*λ*/*A*+*bλ*
*π*_2_	*nλ*(2*n*_0_+1)/*A*(2*n*+3)	*nλ*(2*n*_1_+1)/*A*(2*n*+3)	*nλ*(2*n*_2_+1)/*A*(2*n*+3)	*nλ*/*A*
*π*_3_	*nλ*(2*n*_0_+1)/2*A*(*n*+1)	*nλ*(2*n*_1_+2*n*_2_+1)(2*n*_1_+1)/4*A*(*n*+1)(*n*_1_+*n*_2_+1)	*nλ*(2*n*_1_+2*n*_2_+1)(2*n*_2_+1)/4*A*(*n*+1)(*n*_1_+*n*_2_+1)	*nλ*/*A*
*π*_4_	*nλ*(2*n*_0_+2*n*_2_+1)(2*n*_0_+1)/4*A*(*n*+1)(*n*_0_+*n*_2_+1)	*nλ*(2*n*_1_+1)/2*A*(*n*+1)	*nλ*(2*n*_0_+2*n*_2_+1)(2*n*_2_+1)/2*A*(*n*+1)(*n*_0_+*n*_2_+1)	*nλ*/*A*

**Table 2:** MSEs, ALs, and CPs of *θ*_0_,  *θ*_1_,  *θ*_2_,  and *θ* (*n* = 10).

Method		Para.	*θ*_0_	*θ*_1_	*θ*_2_	*θ*
MLE		MSE	0.4858	0.8030	0.4865	0.9374
		Boot-AL	2.2414	2.7146	2.2266	1.7920
		Boot-CP	0.9339	0.9294	0.9405	0.9321

Bayes	*π*_1_	MSE	0.4850	0.8012	0.4857	0.9340
		HPD-AL	2.0388	2.5119	2.0425	1.9018
		HPD-CP	0.9663	0.9440	0.9645	0.9369
	*π*_2_	MSE	0.4055	0.5903	0.4061	0.9374
		HPD-AL	1.7980	2.2183	1.8016	1.9034
		HPD-CP	0.9552	0.9399	0.9539	0.9335
	*π*_3_	MSE	0.4678	0.5732	0.3909	0.9374
		HPD-AL	1.8797	2.2193	1.7850	1.9034
		HPD-CP	0.9481	0.9405	0.9569	0.9460
	*π*_4_	MSE	0.3748	0.7042	0.3754	0.9374
		HPD-AL	1.7724	2.3192	1.7760	1.9034
		HPD-CP	0.9527	0.9468	0.9515	0.9405

**Table 3:** MSEs, ALs, and CPs of *θ*_0_,  *θ*_1_,  *θ*_2_,  and *θ* (*n* = 20).

Method		Para.	*θ*_0_	*θ*_1_	*θ*_2_	*θ*
MLE		MSE	0.2505	0.4519	0.2523	0.6907
		Boot-AL	1.5795	1.9048	1.5807	1.2957
		Boot-CP	0.9488	0.9483	0.9412	0.9407

Bayes	*π*_1_	MSE	0.2503	0.4512	0.2520	0.6893
		HPD-AL	1.4434	1.7573	1.4382	1.3635
		HPD-CP	0.9832	0.9692	0.9831	0.9415
	*π*_2_	MSE	0.2335	0.3766	0.2350	0.6907
		HPD-AL	1.3512	1.6476	1.3462	1.3640
		HPD-CP	0.9746	0.9447	0.9762	0.9409
	*π*_3_	MSE	0.2551	0.3662	0.2293	0.6907
		HPD-AL	1.3834	1.6486	1.3398	1.3640
		HPD-CP	0.9668	0.9506	0.9777	0.9598
	*π*_4_	MSE	0.2201	0.4260	0.2216	0.6907
		HPD-AL	1.3399	1.6868	1.3348	1.3640
		HPD-CP	0.9614	0.9525	0.9613	0.9498

**Table 4:** MSEs, ALs, and CPs of *θ*_0_,  *θ*_1_,  *θ*_2_,  and *θ* (*n* = 30).

Method		Para.	*θ*_0_	*θ*_1_	*θ*_2_	*θ*
MLE		MSE	0.1752	0.3345	0.1771	0.6049
		Boot-AL	1.2849	1.5451	1.2896	1.0510
		Boot-CP	0.9651	0.9516	0.9654	0.9415

Bayes	*π*_1_	MSE	0.1751	0.3341	0.1770	0.6040
		HPD-AL	1.1710	1.4354	1.1727	1.1164
		HPD-CP	0.9919	0.9629	0.9901	0.9427
	*π*_2_	MSE	0.1694	0.2922	0.1712	0.6049
		HPD-AL	1.1197	1.3745	1.1213	1.1167
		HPD-CP	0.9839	0.9723	0.9835	0.9418
	*π*_3_	MSE	0.1814	0.2849	0.1679	0.6049
		HPD-AL	1.1377	1.3750	1.1177	1.1167
		HPD-CP	0.9783	0.9770	0.9851	0.9615
	*π*_4_	MSE	0.1612	0.3228	0.1629	0.6049
		HPD-AL	1.1132	1.3966	1.1148	1.1167
		HPD-CP	0.9896	0.9581	0.9885	0.9638

**Table 5:** MSEs, ALs, and CPs of *θ*_0_,  *θ*_1_,  *θ*_2_,  and *θ* (*n* = 50).

Method		Para.	*θ*_0_	*θ*_1_	*θ*_2_	*θ*
MLE		MSE	0.1158	0.2460	0.1161	0.5380
		Boot-AL	0.9947	1.1981	1.0018	0.8227
		Boot-CP	0.9829	0.9578	0.9831	0.9554

Bayes	*π*_1_	MSE	0.1157	0.2458	0.1161	0.5375
		HPD-AL	0.9118	1.1075	0.9071	0.8677
		HPD-CP	0.9955	0.9822	0.9954	0.9724
	*π*_2_	MSE	0.1150	0.2243	0.1154	0.5380
		HPD-AL	0.8874	1.0786	0.8828	0.8679
		HPD-CP	0.9884	0.9900	0.9870	0.9702
	*π*_3_	MSE	0.1209	0.2196	0.1137	0.5380
		HPD-AL	0.8961	1.0791	0.8811	0.8679
		HPD-CP	0.9813	0.9933	0.9901	0.9721
	*π*_4_	MSE	0.1105	0.2417	0.1109	0.5380
		HPD-AL	0.8842	1.0892	0.8796	0.8679
		HPD-CP	0.9938	0.9789	0.9931	0.9717

**Table 6:** The simulated data when *n* = 25.

(0.0019	1)	(0.0025	1)	(0.0062	1)	(0.0361	0)	(0.0651	2)	(0.0675	1)	(0.1108	2)	(0.1447	1)	(0.1509	1)	(0.1694	2)	(0.1737	0)	(0.1859	1)	(0.1900	2)	(0.2218	0)	(0.2307	2)	(0.2537	1)	(0.2558	2)	(0.2734	2)	(0.2750	0)	(0.3349	1)	(0.3528	1)	(0.3790	0)	(0.3824	2)	(0.3875	1)	(0.5336	1)

**Table 7:** Point estimates and 95% CIs of *θ*_0_,  *θ*_1_,  *θ*_2_,  and *θ*.

Method		Para.	*θ*_0_	*θ*_1_	*θ*_2_	*θ*
MLE		MLE	0.8029	1.9270	1.2847	4.0146
		Boot-CI	(0.0408, 1.8099)	(0.1891, 2.8803)	(0.0642, 1.8178)	(0.7671, 4.4112)

Bayes	*π*_1_	Bayes	0.8029	1.9267	1.2845	4.0141
		HPD CI	(0.0898, 1.6991)	(0.3473, 2.9083)	(0.0396, 1.7374)	(0.8141, 4.1876)
	*π*_2_	Bayes	0.8332	1.8937	1.2877	4.0146
		HPD CI	(0.0694, 1.7457)	(0.3070, 2.8296)	(0.0364, 1.7697)	(0.7893, 4.4378)
	*π*_3_	Bayes	0.8492	1.8841	1.2812	4.0146
		HPD CI	(0.0798, 1.7561)	(0.1251, 2.5045)	(0.0315, 1.4392)	(0.6368, 4.1407)
	*π*_4_	Bayes	0.8189	1.9301	1.2656	4.0146
		HPD-CP	(0.0638, 1.4011)	(0.2448, 2.8554)	(0.0456, 1.7418)	(0.8474, 4.3484)

**Table 8:** Point estimates and 95% CIs of *θ*_0_,  *θ*_1_,  *θ*_2_,  and  *θ*.

Method		Para.	*θ*_0_	*θ*_1_	*θ*_2_	*θ*
MLE		MLE	1.0882e-2	0.4664e-2	1.3214e-2	2.8760e-2
		Boot-CI	(0.7354e-3, 1.2770e-2)	(0.3244e-2, 2.4914e-2)	(0.4077e-3, 1.5990e-2)	(0.7324e-2, 3.5454e-2)

Bayes	*π*_1_	Bayes	1.1882e-2	0.6879e-2	1.3758e-2	3.2520e-2
		HPD CI	(0.2305e-2, 1.1210e-2)	(0.3998e-2, 1.9825e-2)	(0.1861e-2, 1.4366e-2)	(1.0088e-2, 3.8182e-2)
	*π*_2_	Bayes	1.0832e-2	0.4856e-2	1.3073e-2	2.8760e-2
		HPD CI	(0.8317e-3, 1.1676e-2)	(0.2315e-2, 1.8702e-2)	(0.8807e-3, 1.3700e-2)	(0.5246e-2, 3.2002e-2)
	*π*_3_	Bayes	1.0974e-2	0.4817e-2	1.2969e-2	2.8760e-2
		HPD CI	(0.8499e-3, 1.1535e-2)	(0.3160e-2, 1.7152e-2)	(0.8814e-3, 1.4144e-2)	(0.6994e-2, 3.2140e-2)
	*π*_4_	Bayes	1.0803e-2	0.4919e-2	1.3038e-2	2.8760e-2
		HPD-CP	(0.8949e-3, 1.1372e-2)	(0.3365e-2, 1.8866e-2)	(0.6968e-3, 1.4164e-2)	(0.7224e-2, 3.0467e-2)

## Data Availability

The data used to support the findings of the study are available within the article.

## References

[1] Wang L., Tripathi Y. M., Lodhi C., Zuo X. (2022). Inference for constant-stress Weibull competing risks model under generalized progressive hybrid censoring. *Mathematics and Computers in Simulation*.

[2] Ren J., Gui W. (2021). Statistical analysis of adaptive type-II progressively censored competing risks for Weibull models. *Applied Mathematical Modelling*.

[3] Qin X., Gui W. (2020). Statistical inference of Burr-XII distribution under progressive Type-II censored competing risks data with binomial removals. *Journal of Computational and Applied Mathematics*.

[4] Guan Q., Tang Y., Xu A. (2019). Objective Bayesian analysis for competing risks model with Wiener degradation phenomena and catastrophic failures. *Applied Mathematical Modelling*.

[5] Zhang X. P., Shang J. Z., Chen X., Zhang C. H., Wang Y. S. (2014). Statistical inference of accelerated life testing with dependent competing failures based on copula theory. *IEEE Transactions on Reliability*.

[6] Zhang F., Shi Y., Wang R. (2017). Geometry of the *q*-exponential distribution with dependent competing risks and accelerated life testing. *Physica A: Statistical Mechanics and Its Applications*.

[7] Wang Y., Yan Z. (2021). Statistical inference on accelerated life testing with dependent competing failure model under progressively type II censored data based on copula theory. *Quality and Reliability Engineering International*.

[8] Wu M., Shi Y. M., Zhang C. F. (2017). Statistical analysis of dependent competing risks model in accelerated life testing under progressively hybrid censoring using copula function. *Communications in Statistics - Simulation and Computation*.

[9] Lo S. M. S., Wilke R. A. (2010). A copula model for dependent competing risks. *Journal of the Royal Statistical Society: Series C (Applied Statistics)*.

[10] Fang G., Pan R., Hong Y. (2020). Copula-based reliability analysis of degrading systems with dependent failures. *Reliability Engineering & System Safety*.

[11] Marshall A. W., Olkin I. (1967). A multivariate exponential distribution. *Journal of the American Statistical Association*.

[12] Li Y., Sun J., Song S. (2012). Statistical analysis of bivariate failure time data with Marshall-Olkin Weibull models. *Computational Statistics & Data Analysis*.

[13] Kundu D., Gupta A. K. (2013). Bayes estimation for the Marshall-Olkin bivariate Weibull distribution. *Computational Statistics & Data Analysis*.

[14] Bai X., Shi Y., Ng H. K. T., Liu Y. (2020). Inference of accelerated dependent competing risks model for Marshall-Olkin bivariate Weibull distribution with nonconstant parameters. *Journal of Computational and Applied Mathematics*.

[15] Guan Q., Tang Y., Xu A. (2013). Objective Bayesian analysis for bivariate Marshall-Olkin exponential distribution. *Computational Statistics & Data Analysis*.

[16] Ismail A. A. (2010). Bayes estimation of Gompertz distribution parameters and acceleration factor under partially accelerated life tests with Type-I censoring. *Journal of Statistical Computation and Simulation*.

[17] Ghitany M. E., Alqallaf F., Balakrishnan N. (2014). On the likelihood estimation of the parameters of Gompertz distribution based on complete and progressively Type-II censored samples. *Journal of Statistical Computation and Simulation*.

[18] Bernardo J. M. (1979). Reference posterior distributions for bayesian inference. *Journal of the Royal Statistical Society: Series B*.

[19] Berger J. O., Bernardo J. M., Bernardo J. M. (1992). On the development of reference priors (with discussion). *Bayesian Analysis IV*.

[20] Jeffreys H. (1961). *Theory of Probability*.

[21] Datta G. S., Ghosh M. (1995). Some remarks on noninformative priors. *Journal of the American Statistical Association*.

[22] Chen M.-H., Shao Q.-M. (1999). Monte Carlo estimation of Bayesian credible and HPD intervals. *Journal of Computational & Graphical Statistics*.

[23] Wang L., Tripathi Y. M., Dey S., Shi Y. (2021). Inference for dependence competing risks with partially observed failure causes from bivariate Gompertz distribution under generalized progressive hybrid censoring. *Quality and Reliability Engineering International*.

